# Clinicopathological Risk Factors for Contralateral Lymph Node Metastases in Intraoral Squamous Cell Carcinoma: A Study of 331 Cases

**DOI:** 10.3390/curroncol28030175

**Published:** 2021-05-14

**Authors:** Christian Flörke, Aydin Gülses, Christina-Randi Altmann, Jörg Wiltfang, Henning Wieker, Hendrik Naujokat

**Affiliations:** Department of Oral and Maxillofacial Surgery, Christian Albrechts University, UKSH, 24105 Kiel, Germany; christian.floerke@uksh.de (C.F.); randi_altmann@gmx.de (C.-R.A.); joerg.wiltfang@uksh.de (J.W.); henning.wieker@uksh.de (H.W.); hendrik.naujokat@uksh.de (H.N.)

**Keywords:** squamous cell carcinoma, intraoral, metastasis, contralateral

## Abstract

The current study aimed to examine the effects of clinicopathological factors, including the region, midline involvement, T classification, histological grade, and differentiation of the tumor on the rate of contralateral lymph node metastasis for oral squamous cell carcinoma and to assess their effects on survival rates. A total of 331 patients with intraoral squamous cell carcinomas were included. The influence of tumor location, T status, midline involvement, tumor grading, and the infiltration depth of the tumor on the pattern of metastasis was evaluated. Additionally, the effect of contralateral metastases on the prognosis was examined. Metastases of the contralateral side occurred most frequently in squamous cell carcinomas of the palate and floor of the mouth. Furthermore, tumors with a high T status resulted in significantly higher rates of contralateral metastases. Similarly, the midline involvement, tumor grading, existing ipsilateral metastases, and the infiltration depth of the tumor had a highly significant influence on the development of lymph node metastases on the opposite side. Oral squamous cell carcinomas require a patient-specific decision. There is an ongoing need for further prospective studies to confirm the validity of the prognostic factors described herein.

## 1. Introduction

Lymph node involvement by all malignancies increases the risk of recurrence and mortality. Annually, 200,000–350,000 new cases of oral SCC involving the floor of the mouth, followed by the inner cheek, alveolar process, hard palate, anterior two-thirds of the tongue, anterior one-third of the soft palate, and the non-keratinized part of the lip were reported [1,2]. A 5-year survival rate for oral SCC was reported to be 54.6% [3], where one-third of all lymph nodes in the human organism are located in the head and neck region [4]. Furthermore, 80% of oral SCCs were diagnosed at advanced stages (T2–T4) [5,6], and in 50% of patients, the lymph nodes are already unilaterally or bilaterally affected at the time of initial diagnosis [7,8] and the risk of occult lymph node involvement is suggested to be higher than 20% [9,10].

In the literature, the information regarding the need for a contralateral lymph node dissection in intraoral SCCs is limited. Kowalski et al. have described a mathematical model for the risk assessment of contralateral neck dissections in oral SCC and proclaimed that not all tumors crossing midline are associated with a high risk (stages I and II tumors not involving the floor of the mouth) and not all tumors without midline involvement are at low risk (stages III and IV tumors involving the floor of the mouth) [11]. Kurita et al. [12] reported that patients with advanced tumors, multi-involvement of the ipsilateral neck nodes, or a higher degree of histopathological grading were at a higher risk for developing contralateral lymph node metastases. However, due to the varying metastatic lymph node metastasis patterns of oral SCCs, there is still a need for studies describing the influencing factors. Therefore, the current retrospective study aimed to examine the effects of various clinicopathological factors on the rate of contralateral lymph node metastasis of oral SCCs and to assess their effects on survival rates.

## 2. Materials and Methods

This retrospective study was approved by the Ethical Commission of the Christian Albrechts University, Faculty of Medicine. (D 710/21)

### 2.1. Study Design

Data of patients who underwent a lymphadenectomy between 2006 to 2012 due to an intraoral SCC were collected via the information system Orbis (Agfa Health Care GmbH, Bonn, Germany) regarding the OPS codes.

The inclusion criteria were:

- Histologically confirmed that squamous cell carcinoma must be the primary tumor.
- Tumor must have been primarily treated surgically.
- The localization of the intraoral squamous epithelium must be limited to the floor of the mouth, tongue, alveolar process, hard palate, inner cheek, parotid gland, oropharynx, or lip.
- The initial diagnosis must have been made in the years between 2007–2012.

Patients who received primarily radiotherapy and had other malignancies of the oral cavity were excluded.

Information regarding the TNM status depth of infiltration and the resection status were obtained from the histopathological reports. For the generation of information on systemic diseases, tumor location, and surgical technique, operation reports were used. The day of surgical resection of the primary tumor was taken as the starting point for calculating the survival interval. The last vital date was defined as the end of the survival time. The time of tumor recurrence (local recurrence, lymph node recurrence, distant metastases) and secondary tumors were recorded as follow-up data in the context of follow-up examinations. The disease-free survival (DFS) was determined and all information was anonymized and transferred to an Excel database.

### 2.2. Lymphadenectomy

In the current study, the nomenclature developed by Robbins et al. was used for the description of cervical lymph nodes [13]. According to this nomenclature, six levels were described, with levels I, II, and V each being assigned sublevels A and B. As a standard procedure, selective lymph node dissections were performed supra-omohyoidally at levels I–III.

### 2.3. Statistical Analysis

SPSS (IBM, SPSS Statistics, Version 24.0 for Mac) was used. Frequency tables, crosstabs, bar and pie charts, and histograms were used to represent the descriptive statistics. Relationships between different characteristics of samples were determined using cross tables. The probabilities of the connections were checked by using the chi-square test. The mean value differences of two independent groups were determined using Student’s *t*-test. A significance level of *p* ≤ 0.05 was set for all statistical analyses. The survival of the patients was analyzed and determined via Kaplan–Meyer curves.

## 3. Results

### 3.1. Demographic Data

The data were from 350 patients who were recruited with a diagnosis of “squamous cell carcinoma of the head and neck region” from January 2006 until December 2012. The extraoral manifestations observed by 19 patients were excluded. A total of 331 patients (226 men (4.8%) and 124 women (35.2%) with a mean age of 63.71 ± 21.17 years) with intraoral squamous cell carcinomas were included.

### 3.2. Tumor Localization

Distribution regarding tumor localization showed that, among the 350 documented cases, the localization sites of the primary tumor were as follows:

- Extraoral manifestation, *n* = 19 (5.4%)
- Floor of the mouth, *n* = 99 (28.3%)
- Tongue, *n* = 74 (21.1%)
- Mandibular alveolar process, *n* = 73 (20.9%)
- Lower lip, *n* = 31 (8.9%)
- Maxillary alveolar process, *n* = 19 (5.4%)
- Regio buccalis, *n* = 20 (5.7.0%)
- Soft and hard palate, *n* = 14 (4.0%)
- Oropharynx, *n* = 1 (0.3%)

### 3.3. T-Status (T) and Midline Involvement

The T stages of the carcinomas in situ—T1, T2, T3, and T4 (*n_total_* = 350 patients)—were documented. In five patients, the T status could not be determined due to documentation failure and were excluded. In addition, data of patients with “carcinoma in situ” (*n* = 8) were not included in the T stage analysis. Among 345 patients, T1 was the most frequently recorded status (*n* = 132, 39.2%), followed by T2 (*n* = 95, 28.2%), T3 (*n* = 50, 14.8%), and T4 (*n* = 60, 17.8%). Analysis of the midline involvement revealed that, in 82 cases (23.4%), the tumor exceeded the midline, whereas no involvement of the midline was observed in 249 (75.2%) cases.

### 3.4. Lymph Node Status (N) and Neck Dissections

Neck dissection was performed on a total of 346 patients (98.9%). Lymphadenectomy was limited to levels I–III in 249 cases (71.1%) on the right and in 253 cases on the left (72.3%) sides. Levels I–V dissections were also performed by 97 patients (27.7%) on the right and 96 patients (27.4%) on the left sides. In four cases, selective lymph node picking was performed.

**Figure 1:**
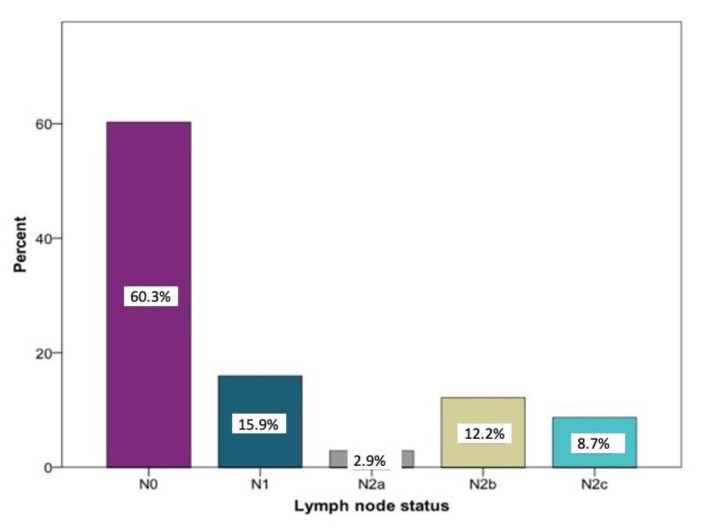
Distribution of the lymph node stages N0 (*n* = 208), N1 (*n* = 55), N2a (*n* = 10), N2 b (*n* = 42), and N2c (*n* = 30) according to histopathological examinations relative to the study sample (*n* = 350) in percent.

Overall, the histopathological lymph node staging was carried out in 350 patients. N0 was determined in 208 cases (60.3%). A total of 142 patients (40.6%) had positive lymph node findings. Among these, N1 the was most frequently documented (15.9%) stage. Larger metastases occurred more frequently in several regional lymph nodes on the ipsilateral side: stage N2a was registered 10 times (2.9%) and stage N2b was registered 42 times (12.2%). Bilateral or contralateral metastases were found in 30 patients (8.7%) (N2c). In five patients, no information regarding the histopathological lymph node status could be found (Figure 1).

**Figure 2:**
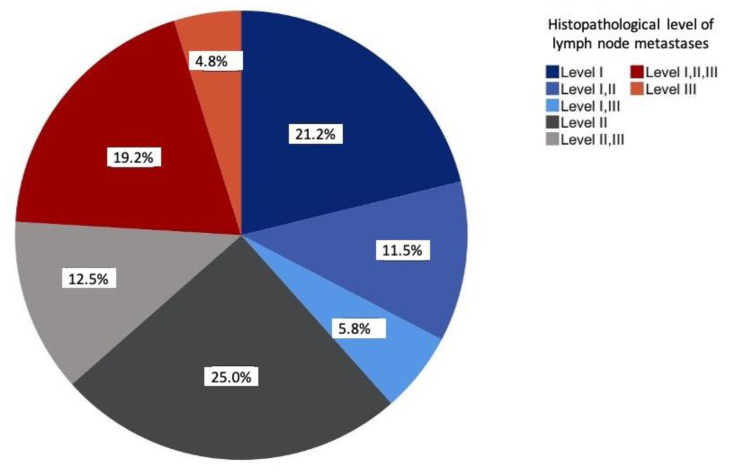
Distribution of the lymph node metastases according to histopathological examinations relative to the study sample (*n* = 104) in percent.

The prevalence of solitary metastases was most frequently seen at level II (25%), followed by level I (20.2%). Overall, more than half of all lymph node metastases occurred at levels I and II (57.7%). The involvement of levels I–III was observed in 19.2% of the cases. At 4.8%, level III was the least solitarily affected level (Figure 2).

### 3.5. Analysis of the Tumor Localization and Lymph Node Metastases

**Figure 3:**
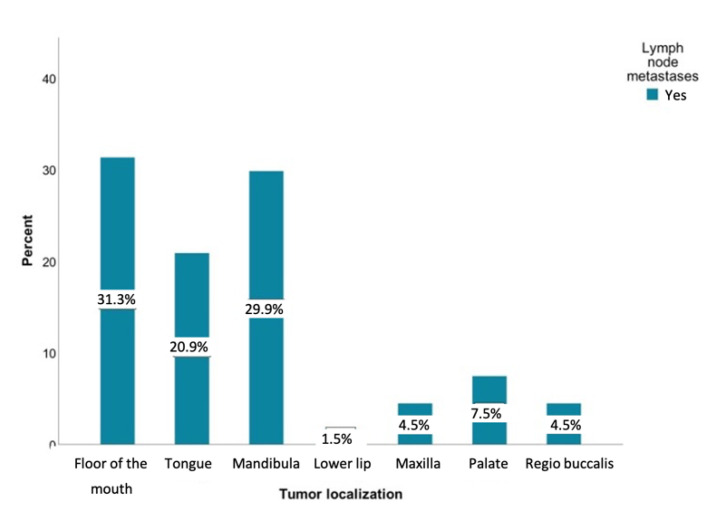
Distribution of patients with lymph node metastases regarding the tumor sites relative to the study sample (*n* = 138) in percent: floor of the mouth (*n* = 42), tongue (*n* = 28), lower jaw (*n* = 40), lower lip (*n* = 2), upper jaw (*n* = 6), palate (*n* = 10), and inner cheek (*n* = 6).

Metastases were most commonly observed in patients with tumors involving the floor of the mouth (*n* = 42, 30.4%), mandibular alveolar process (*n* = 40, 29%), and the tongue (*n* = 28, 20.3%). It is remarkable that tumors of the lower lip developed almost no metastases (*n* = 42, 1.4%). Figure 3 shows the distribution of lymph node metastases within different tumor sites.

**Figure 4:**
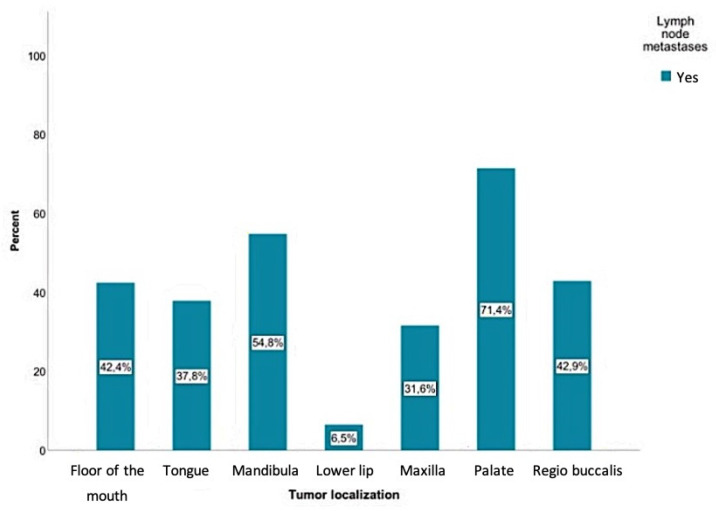
Positive lymph node findings were mostly detected in SCCs involving the palate (71.4%), followed by the mandible (54.8%), the inner cheek *(*regio buccalis*)* (42.9%), the floor of the mouth (42.4%), and the tongue (37.8%). A total of 9300 lymph nodes were removed, where 384 of those were detected as positive for a tumor.

The current study showed that positive lymph node findings were mostly detected in SCCs involving the palate (71.4%), followed by the mandible (54.8%), the inner cheek (regio buccalis) (42.9%), the floor of the mouth (42.4%), and the tongue (37.8%). Regarding tumors of the mandibular alveolar process, palate, and oropharynx, a positive lymph node finding was even more common than a negative one. The probability of positive lymph node findings for each tumor location is shown in Figure 4. According to the chi-square test, there was a significant relationship between the tumor location and the presence of lymph node metastases (*p* < 0.01).

**Figure 5:**
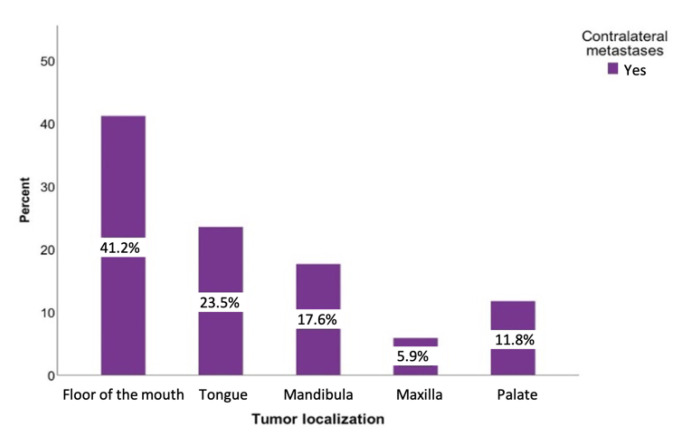
Distribution of the tumor-positive contralateral lymph nodes regarding the tumor sites: floor of the mouth, tongue, lower jaw, lower lip, upper jaw, and palate.

A total of 107 patients (34.6%) had metastases on the ipsilateral side. The contralateral side was affected in 35 cases (8.6%). Regarding the contralateral metastases, the floor of the mouth was the region where contralateral metastases (51.43%) were most commonly found. The tumors of the buccal plane, lower lip, and oropharynx never led to a positive contralateral lymph node finding (Figure 5).

**Figure 6:**
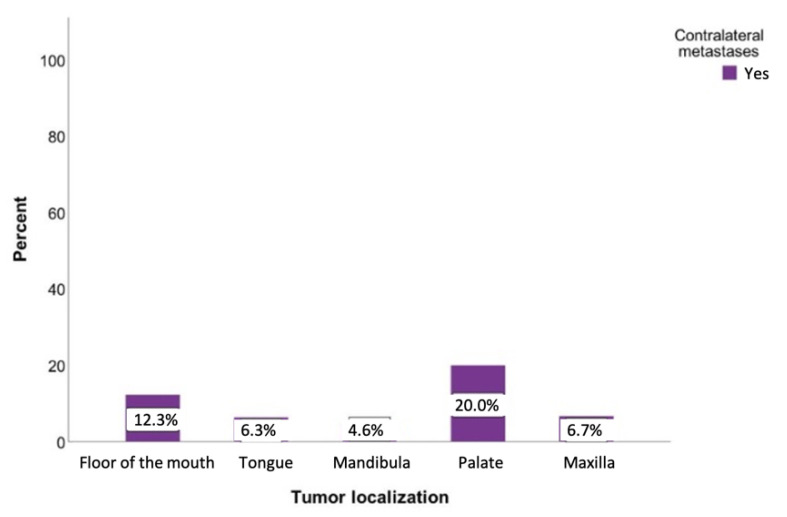
Possibility of developing contralateral lymph node metastases regarding the tumor localization. Total for each *x*-axis category: floor of the mouth (*n* = 57), tongue (*n* = 63), lower jaw (*n* = 65), lower lip (*n* = 17), upper jaw (*n* = 15), palate (*n* = 10), inner cheek (*n* = 15), parotid (*n* = 6), and oropharynx (*n* = 1).

Tumors originating from the palate had the highest probability of developing contralateral metastases at 20%. If the tumor was located on the floor of the mouth, there was also an increased likelihood of developing metastases on the opposite side (12.3%). This probability was slightly lower for tumors of the tongue and the upper jaw (6.4% and 6.8%, respectively) (Figure 6). However, a chi-square analysis revealed a statistically insignificant relationship between the tumor location and contralateral metastases (*p* = 0.097).

### 3.6. Analysis of the T Classification and Lymph Node Metastases

**Figure 7:**
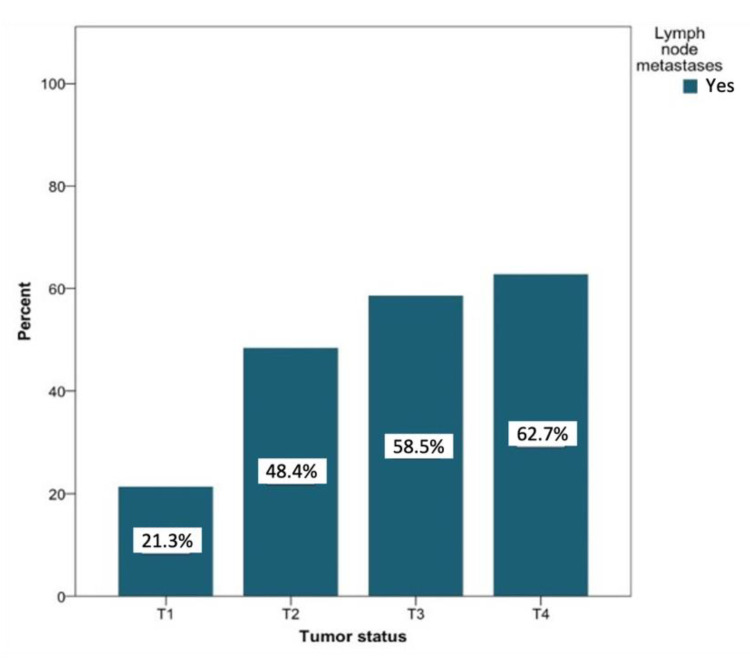
Distribution (in percent) of tumor-positive lymph node findings depending on the tumor status. Total for each *x*-axis category: T1 (*n* = 127), T2 (*n* = 91), T3 (*n* = 41), and T4 (*n* = 59).

In patients with lymph node metastases that were secondary to T1 tumors (*n* = 127), negative lymph node findings were more common (31.9% vs. 8.8% for positive findings). Patients with lymph node metastases secondary to T2 tumors (*n* = 91) showed about the same probability of developing metastases (14.7%) compared to negative findings (15.1%). Among patients with positive lymph node findings of tumor status with classifications T3 and T4, a positive lymph node finding was recorded more often than negative lymph nodes (6.3% vs. 5% and 10.95 vs. 7.1%, respectively). Figure 7 shows the likelihood of the development of metastases based on the tumor status. This probability increased with increasing T status (T1 to T4) from 21.3% to 62.7%. The relationship between the tumor status and the presence of lymph node metastases was significant at the level of *p* < 0.01.

**Figure 8:**
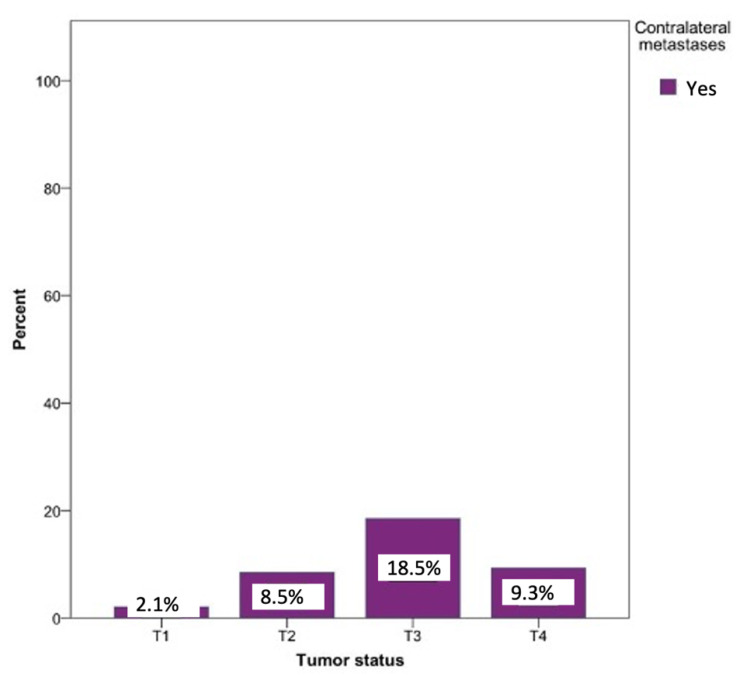
Possibility of developing contralateral lymph node metastases depending on the tumor localization. Total for each *x*-axis category: floor of the mouth (*n* = 57), tongue (*n* = 63), lower jaw (*n* = 65), lower lip (*n* = 17), upper jaw (*n* = 15), palate (*n* = 10), inner cheek (*n* = 15), parotid (*n* = 6), and oropharynx (*n* = 1).

Metastases on the opposite side could be observed in every tumor status. The probability of developing contralateral metastases was significantly lower in the T1 (0.8%), T2 (2.5%), and T4 (1.7%) tumor statuses. Contralateral metastases were most commonly seen in patients with a T3 tumor status (18.5%). Surprisingly, T4 status tumors revealed a 9.3% lower percentage of contralateral metastases compared to T3. The relationship between the tumor status and the likelihood of developing a contralateral metastasis was statistically significant (*p* = 0.03) (Figure 8).

### 3.7. Analysis of the Midline Involvement and Lymph Node Metastases

A total of 50% of all contralateral metastases were diagnosed in patients whose primary tumor exceeded the midline (*n* = 17). The relationship between midline involvement and the development of contralateral metastases was statistically highly significant (*p* = 0.00) (Table 1).

### 3.8. Analysis of the Depth of the Tumor Infiltration and Lymph Node Metastases

The mean infiltration depth within the entire patient collective was 7.00 mm. The influence of the infiltration depth on the lymph node metastasis revealed that the mean value of infiltration depth in patients with lymph node metastases (*n* = 40) was 9.1 mm. Among patients without lymph node findings (*n* = 77), the mean infiltration depth was found to be 5.9 mm. The relationship between the depth of the tumor infiltration and lymph node metastases was significant according to Spearman’s rho at the level of *p* = 0.01. In patients with contralateral metastases (*n* = 7), the mean value of the infiltration depth was 10.9 mm. According to the Spearman rho analysis, this relationship was statistically significant (*p* = 0.05).

### 3.9. Contralateral vs. Ipsilateral Metastases

Assessment of the relationship between existing ipsilateral metastases and the development of contralateral metastases revealed that, in the majority of the cases (*n* = 12), contralateral metastases occurred with existing ipsilateral metastases. In five cases, contralateral metastases were observed without ipsilateral metastases. The likelihood of developing a contralateral metastasis given the absence of ipsilateral metastases was found to be 3.1%. If metastases already existed on the side of the primary tumor, this probability increased up to 13.6%. Comparative analysis of this influence was statistically significant (*p* = 0.002).

### 3.10. Preoperative CT Findings and Histopathological Analysis of the Metastases

According to the clinical standards, all patients underwent a staging procedure prior to therapy planning, which involved a CT of the head and neck, and thorax and abdomen ultrasonography. The data showed that, in 78.5% of patients with surgically approved lymph node metastases, the preoperative CT was in accordance with histological findings. However, in 21.6% of the cases, metastases could not be detected in the CT. For 42.4% of the patients, the lymph node findings were detected as a false positive, which means that despite suspicious lymph nodes in the CT, no lymph node metastases could be diagnosed histopathologically.

### 3.11. Five-Year-Survival and Disease-Free-Survival Rates

**Figure 9:**
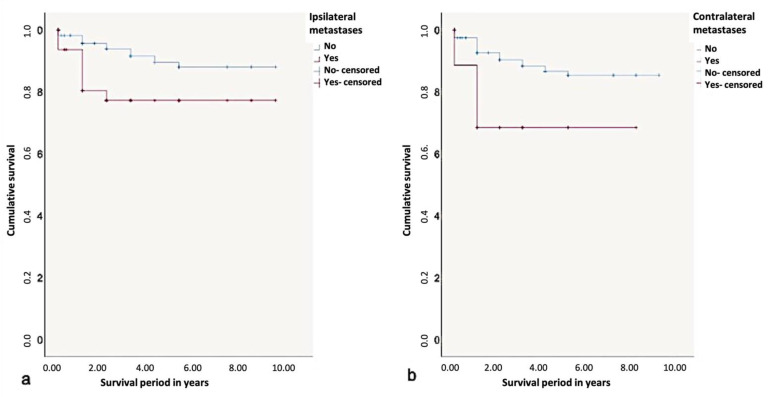
Kaplan-Meier survival curves for survival period in years regarding (**a**) ipsi- and (**b**) contralateral metastases.

The 5-year-survival rate without the influence of a prognostic factor was found to be 90%. Depending on the influence of ipsilateral metastases, it could decrease to 75%. Considering the metastasis patterns, the 5-year-survival rate was found to be the lowest with the presence of contralateral metastases (65%). Contralateral metastases had a significant influence on survival rates according to the log-rank test (*p* < 0.01) (Figure 9).

**Figure 10:**
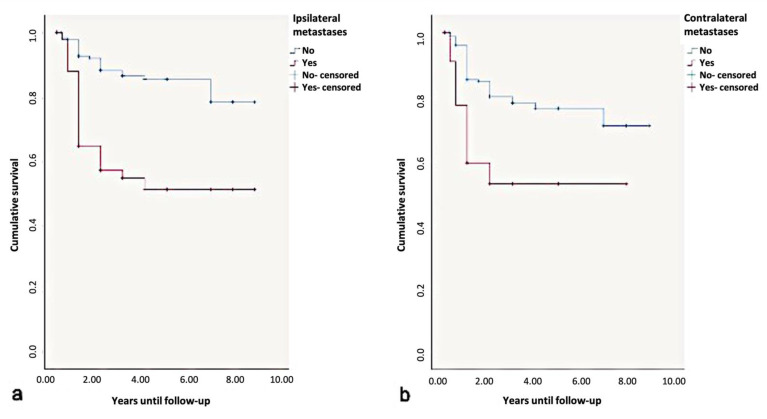
Kaplan-Meier survival curves for survival period in disease-free. years regarding (**a**) ipsi- and (**b**) contralateral metastases.

Overall, the 5-year disease-free-survival rate was found to be 85%. Due to the influence of ipsi- or contralateral metastases, the 5-year disease-free=survival rate dropped to 50%. According to the log-rank test, contralateral metastases significantly influenced the disease-free-survival rates (*p* < 0.01) (Figure 10).

## 4. Discussion

The main focus of the current work was to examine the factors influencing the contralateral metastasis of oral SCCs and determine the need for a contralateral elective neck dissection.

The prevalence of metastases in levels I and II observed in the current study was in accordance with the existing literature [11,14]. A small proportion of metastases were exclusively diagnosed at level III (4.8%) and coincided with the data of previous studies [14,15]. Combined metastases at levels I–III (37.5%) were found for all primary tumor sites, except the tongue.

The presence of metastases in levels IV and V is considered to be a crucial factor for patient survival [16]. Kowalski et al. reported a 1.4% probability of developing lymph node metastases in levels IV and V for patients with oral SCC and lymph node status pN1 [15]. For tumors of the floor of the mouth, tongue, and lower jaw, the probability was found to be higher in the current study. The results described herein also showed that SCC of the tongue could result in level IV metastases with a chance of 7.4%. Wolgar et al. recommended that, due to the risk of involvement of level IV, therapeutic neck dissection for SCC of the tongue should be extended [14].

The current study showed a statistically significant relationship between metastasis behavior and tumor location (*p* < 0.01). With all primary tumors, except those of the lip, the probability of developing lymph node metastases was over 30%. SCCs of the palate led to metastases in 71% of cases. Tumors that originated on the floor of the mouth, mandibular alveolar process, and buccal plane had a 40% or higher probability of developing metastases.

The floor of the mouth as the location of the primary tumor is often associated with a high probability of metastasis in the literature. Iype et al. [15] observed a 35% probability of developing metastases for clinically diagnosed N0 patients in whom the tumors were located on the floor of the mouth. For primary tumors of the buccal plane, tongue, and alveolar process of the mandible, the likelihood of developing metastases was found to be 25–26% [15]. In addition to tumors of the floor of the mouth, Remmert et al. also found a higher ratio of ipsilateral and contralateral metastases for SCC of the lip [17], which particularly differs from the results presented herein.

According to a study by Capote-Moreno et al., SCC of the tongue is most likely (31.4%) to develop contralateral metastases, followed by SCC of the floor of the mouth (11%) [18]. The probability of developing contralateral metastases secondary to the SCC of the floor of the mouth was found to be slightly higher with a prevalence of 12.3%. Moreover, the probability of contralateral metastasis due to SCC of the tongue was observed to be 6.4%. Fan et al. stated that contralateral metastasis secondary oral SCCs of the tongue and floor of the mouth could differ from the area affected. For example, the base of the tongue and the floor of the mouth lead to contralateral metastases much more frequently than those of the anterior part of the tongue and retromolar region [18]. In the current study, no sub-classification was made. This might have led to the fact that, in contrast to the existing literature, the tongue (6.4%) posed a lower risk and for developing contralateral metastases, while the mandibular alveolar process posed a higher risk (4.6%).

Overall, the results of the present study were in agreement with previous studies, which stated that the involvement of the floor of the mouth and tongue could be determined as a significant prognostic factor for the development of contralateral metastases [11,19]. It has also been observed that patients whose tumors originate on the palate also have an increased likelihood of developing metastases on the opposite side (20%). Several authors have also recommended bilateral lymph node removal in all cases for tumors of the palate with a tumor status > 1 [20,21,22]. However, in the current study, no statistical significance could be determined between the tumor location and the risk of developing contralateral metastases (*p* = 0.097). This might be explained by the limited number of patients with primary tumors exceeding the midline (*n* = 17).

The influence of tumor size is often described in the literature as one of the most important factors influencing lymph node metastasis [17,19]. With regard to contralateral metastases, a statistical significance between the tumor size and contralateral metastasis (*p* = 0.03) was found. Tumors with statuses T3 and T4 led to contralateral metastases in 18.5% and 9.3% of the cases, respectively. Koo et al. reported a similar distribution and higher probability of 25% for T3 tumors and a lower probability of 18% for T4 tumors for developing contralateral metastases [20]. A possible reason for the lower rate of contralateral metastases in T4 compared to T3 could be the size-related proximity to the midline, which might have led to an exclusion in the analysis of the contralateral metastasis behavior, as described herein. In the current study, 2% of T1 tumors developed contralateral metastases, which was in accordance with previous studies [11,20,21]. Olzowy et al. also highlighted the overestimation of the risk of developing contralateral metastases for small tumors (T1, T2) and an underestimation for those with an advanced status (T3, T4) [22].

Many authors recognize that one of the most important factors influencing the risk of contralateral metastases is the involvement of the midline [20,23]. Kowalski et al. proclaimed that if the tumor exceeds the midline by more than an inch, there is an 8.8-fold higher risk of developing contralateral metastases [11]. In the current study, a total of 80 tumors (24.4%) exceeded the midline, and in 17 cases, contralateral lymph node findings were observed. The significant correlation (*p* < 0.01) between the involvement of the midline and the development of contralateral metastases was in agreement with the existing literature [21].

In this study, 137 patients with oral SCC were histopathologically diagnosed with lymph node metastases. If ipsilateral metastases had already been diagnosed, the risk for developing contralateral metastases was found to be significantly higher (13.6%) than if no metastases were found on the side of the primary tumor (3.1%) (*p* = 0.002). Olzowy et al. observed a significant relationship between the presence of two or more ipsilateral metastases and the development of bilateral metastases [24]. According to Kowalski et al. [11], the risk of developing contralateral metastases is 4.8 times higher if ipsilateral metastases have already been diagnosed.

In the literature, a tumor infiltration depth of more than 3–4 mm is stated to be a decisive factor for the survival and development of lymph node metastases [25,26,27,28]. Regarding contralateral metastases, Bier-Laning et al. proclaimed that infiltration of <3.75 mm never results in lymph node metastases on the opposite side. In addition, this risk increases by 5% per mm [29]. The increasing probability of developing contralateral metastases at higher infiltration depths (>6 mm) described herein was also previously described [11,21,30]. The infiltration depth provides important additional information for tumor staging and the concomitant therapeutic strategies. Furthermore, it should be kept in mind that different tumor sites could show distinct metastatic behavior.

The prognostic relevance of contralateral metastases on survival rates has been evaluated in numerous studies [11,21]. Capote-Moreno et al. found a decrease in the 5-year survival rate from 70% to 41.2% for contralateral metastases [18]. According to Spiro et al., the 5-year survival rate for ipsilateral metastases was significantly lower at 28% and falls again by 5% under the influence of contralateral metastases [31]. The results of the current study revealed that if contralateral metastases were diagnosed, the 5-year-survival rate decreased from 75% to 65%. However, it should be kept in mind that, the prognosis of the condition depends on many factors, including surgical intervention, pathological findings, final staging, and post-operative management, such as radiotherapy, chemotherapy, immunotherapy, or a combination thereof. Due to the variety of factors determining the prognosis, the relationship between the lymph node metastases and survival rates warrants further research.

## 5. Conclusions

Metastases of the contralateral side occur most frequently in SCCs of the palate and floor of the mouth. Furthermore, tumors with a high T status result in significantly higher rates of contralateral metastases. Similarly, the midline involvement, existing ipsilateral metastases, and the infiltration depth of the tumor had a highly significant influence on the development of lymph node metastases on the opposite side.

## Figures and Tables

**Table 1:** Distribution of contralateral metastases regarding the midline involvement.

		Contralateral Metastases		
		−	+	Total
**Midline involvement**	**−**	230	17	247
	**+**	63	17	80
	**Total**	293	34	327

## Data Availability

The data used for the present article are available in research laboratories of CAU, Dept. OMFS.
